# Supplementary material for: Placebo treatment affects brain systems related to affective and cognitive processes, but not nociceptive pain
Source: Nat Commun. 2024 Jul 17;15:6017. doi: 10.1038/s41467-024-50103-8 (PMC11255344; doi:10.1038/s41467-024-50103-8)
Supplement: Supplementary file 1 — Supplementary Information [file 41467_2024_50103_MOESM1_ESM.pdf]

Supplementary Information

Placebo treatment affects brain systems related to affective and cognitive processes, but not nociceptive pain

Rotem Botvinik-Nezer[1,2,*,**], Bogdan Petre[2,*], Marta Ceko[3], Martin A. Lindquist[4], Naomi P. Friedman[5,6], Tor D. Wager[2,**]

[1] Department of Psychology, The Hebrew University of Jerusalem, Jerusalem, Israel; [2] Department of Psychological and Brain Sciences, Dartmouth College, Hanover, NH, USA; [3] Institute of Cognitive Science, University of Colorado Boulder, Boulder, CO, USA; [4] Department of Biostatistics, Johns Hopkins University, Baltimore, MD, USA; [5] Institute for Behavioral Genetics, University of Colorado Boulder, Boulder, CO, USA; [6] Department of Psychology and Neuroscience, University of Colorado Boulder, Boulder, CO, USA;

* These authors contributed equally to this work;

** Corresponding authors (emails: rotemb9@gmail.com; tor.D.Wager@dartmouth.edu)

Supplementary Results

Behavioral results with unpleasantness ratings

Results were quantitatively similar when using the unpleasantness rather than the intensity ratings provided by the participants after each trial. Participants' unpleasantness ratings following noxious stimuli were significantly lower in the Placebo compared to the Control condition (thermal: Placebo M = 0.109, Control M = 0.162, β = -0.375, SE = 0.039, $t_{(234.8)}$ = -9.62, p < .001, 95% CI = [-0.452, -0.298]; mechanical: Placebo M = 0.095, Control M = 0.130, β = -0.233, SE = 0.039, $t_{(363.5)}$ = -5.77, p < .001, 95% CI = [-0.298, -0.147]). As expected, unpleasantness ratings increased with increasing stimulus intensity (thermal: low intensity M = 0.111, medium intensity M = 0.128, high intensity M = 0.168, β = 0.399, SE = 0.031, $t_{(209.4)}$ = 12.70, p < .001, 95% CI = [0.337, 0.461]; mechanical: low intensity M = 0.100, medium intensity M = 0.109, high intensity M = 0.128, β = 0.214, SE = 0.033, $t_{(151.4)}$ = 6.57, p < .001, 95% CI = [0.149, 0.278]). The Intensity X Placebo interaction was not significant (thermal: β = -0.100, SE = 0.053, $t_{(1253.1)}$ = -1.87, p = .062, 95% CI = [-0.204, 0.005]; mechanical: β = 0.013, SE = 0.038, $t_{(1178.3)}$ = 0.35, p = .724, 95% CI = [-0.061, 0.087]).

Robustness to covariates and demographic effects

Pain ratings

The results indicating effects of the placebo condition and the stimulus intensity on pain ratings were robust to the inclusion of demographic covariates, sex and age. To test this, we ran the same models as described in the main text, with sex and age as additional covariates. Since age is the same within twin pairs, and sex is the same for the vast majority (except some opposite-sex dizygotic twins), age was only added as a fixed effect, and sex was added as a fixed effect and a random effect at the family level.

Pain ratings were significantly lower in the Placebo compared to the Control condition (thermal: β = -0.359, SE = 0.037, $t_{(235.5)}$ = -9.72, p < .001, 95% CI = [-0.432, -0.286]; mechanical: β = -0.240, SE = 0.040, $t_{(273.3)}$ = -6.06, p < .001, 95% CI = [-0.317, -0.162]) and significantly higher for higher stimulus intensity (thermal: β = 0.409, SE = 0.031, $t_{(347.8)}$ = 13.22, p < .001, 95% CI = [0.348, 0.470]; mechanical: β = 0.206, SE = 0.031, $t_{(129.4)}$ = 6.53, p < .001, 95% CI = [0.143, 0.268]). Males reported significantly higher pain compared to women in the mechanical (β = 0.187, SE = 0.086,

$t_{(177.1)}$ = 2.18, p = .031, 95% CI = [0.017, 0.356]), but not in the thermal (β = -0.033, SE = 0.075, $t_{(266.16)}$ = -0.44, p = .661, 95% CI = [-0.180, 0.115]) modality, while age did not affect pain ratings in either modality (thermal: β = -0.009, SE = 0.037, $t_{(326.9)}$ = -0.23, p = .816, 95% CI = [-0.082, 0.065]; mechanical: β = 0.058, SE = 0.040, $t_{(207.0)}$ = 1.42, p = .156, 95% CI = [-0.022, 0.137]).

Furthermore, we did not find significant sex differences in placebo analgesia for the thermal pain trials (Control minus Placebo, female: M = 0.054, SD = 0.103, N = 217; male: M = 0.053, SD = 0.102, N = 150; mixed effects model predicting placebo analgesia with fixed and random effects of stimulus level and sex, and a fixed effect of age: β = 0.030, SE = 0.081, $t_{(263.49)}$ = 0.42, p = .706738, 95% CI = [-0.125, 0.194]). The placebo effect was also not significantly related to age (β = 0.073, SE = 0.040, $t_{(263.87)}$ = 1.81, p = .072, 95% CI = [-0.006, 0.153]. These two effects were similarly not significant in the mechanical pain modality (sex: female: M = 0.0.39, SD = 0.121, N = 216; male: M = 0.041, SD = 0.132, N = 152; β = -0.014, SE = 0.094, $t_{(237.41)}$ = -0.15, p = .883, 95% CI = [-0.198, 0.171]; age: β = -0.021, SE = 0.046, $t_{(302.87)}$ = -0.46, p = .649, 95% CI = [-0.111, 0.069]).

NPS

Including gender and age as covariates did not change the NPS results. The NPS score was significantly higher for higher intensity levels (thermal: β = 0.244, SE = 0.041, $t_{(678.9)}$ = 5.99, p < .001, 95% CI = [0.164, 0.324]; mechanical: β = 0.163, SE = 0.041, $t_{(698.6)}$ = 3.98, p < .001, 95% CI = [0.083, 0.243]) and was not significantly different as a function of the placebo condition (thermal: β = -0.031, SE = 0.036, $t_{(225.5)}$ = -0.86, p = .329, 95% CI = [-0.102, 0.040]; mechanical: β = 0.008, SE = 0.037, $t_{(277.74)}$ = 0.21, p = .831, 95% CI = [-0.065, 0.061]). There was a significant main effect of sex, such that the NPS score was significantly higher for females in the thermal (β = -0.200, SE = 0.067, $t_{(336.6)}$ = -2.98, p = .003, 95% CI = [-0.332, -0.068]) but not mechanical (β = -0.063, SE = 0.074, $t_{(187.7)}$ = -0.85, p = .265, 95% CI = [-0.209, 0.083]) pain trials, and there was no effect of age on the NPS score in both modalities (thermal: β = 0.001, SE = 0.034, $t_{(289.1)}$ = 0.04, p = .971, 95% CI = [-0.065, 0.067]; mechanical: β = 0.033, SE = 0.035, $t_{(269.7)}$ = 0.93, p = .356, 95% CI = [-0.037, 0.102]). Furthermore, the placebo-induced NPS reductions still significantly correlated with the behavioral analgesia in both modalities when controlling for age and sex (thermal: β = 0.199, SE = 0.034, $t_{(101.9)}$ = 5.90, p < .001, 95% CI = [0.132, 0.266]; mechanical: β = 0.199, SE = 0.037, $t_{(60.1)}$ = 5.35, p < .001, 95% CI = [0.125, 0.274]).

SIIPS

Similarly to the behavioral and NPS results, the SIIPS results were robust to the inclusion of gender and age as covariates. When including these demographic covariates, the SIIPS score was significantly lower in the Placebo compared to the Control condition in both the thermal (β = -0.128, *SE* = 0.033, $t_{(440.5)}$ = -3.86, p < .001, 95% CI = [-0.194, -0.063]) and mechanical (β = -0.111, *SE* = 0.037, $t_{(272.8)}$ = -2.98, p = .003, 95% CI = [-0.185, -0.038]) modalities. The SIIPS score also increased with stimulus intensity in both modalities (thermal: β = 0.180, *SE* = 0.038, $t_{(1050)}$ = 4.73, p < .001, 95% CI = [0.105, 0.255]; mechanical: β = 0.188, *SE* = 0.040, $t_{(232.4)}$ = 4.76, p < .001, 95% CI = [0.110, 0.266]). The placebo-induced SIIPS reductions significantly correlated with the behavioral analgesia in both modalities also when controlling for age and sex (thermal: β = 0.237, *SE* = 0.033, $t_{(248.3)}$ = 7.08, p < .001, 95% CI = [0.171, 0.303]; mechanical: β = 0.237, *SE* = 0.035, $t_{(86.4)}$ = 6.73, p < .001, 95% CI = [0.167, 0.307]). In addition, the SIIPS score was significantly higher for females compared to males in the thermal (β = -0.190, *SE* = 0.074, $t_{(251.6)}$ = -2.58, p = .010, 95% CI = [-0.336, -0.045]) but not in the mechanical (β = -0.027, *SE* = 0.073, $t_{(192.1)}$ = -0.37, p = .714, 95% CI = [-0.172, 0.118]) pain modality, and there was no effect of age on the SIIPS score in both modalities (thermal: β = -0.012, *SE* = 0.036, $t_{(272.2)}$ = -0.33, p = .745, 95% CI = [-0.083, 0.059]; mechanical: β = -0.003, *SE* = 0.036, $t_{(231.1)}$ = -0.09, p = .931, 95% CI = [-0.075, 0.068]). The placebo-induced SIIPS reduction was stronger for younger participants in the thermal (β = -0.070, *SE* = 0.032, $t_{(418)}$ = -2.20, p = .029, 95% CI = [-0.132, -0.007]) but not in the mechanical (β = 0.018, *SE* = 0.033, $t_{(401.1)}$ = 0.56, p = .576, 95% CI = [-0.046, 0.083]) pain modality, and there were no sex effects on the placebo-induced SIIPS reductions (thermal: β = -0.062, *SE* = 0.065, $t_{(222)}$ = -0.95, p = .341, 95% CI = [-0.190, 0.066]; mechanical: β = -0.104, *SE* = 0.068, $t_{(204.8)}$ = -1.51, p = .132, 95% CI = [-0.239, 0.031]).

Additional, non-pre-registered SIIPS subregions

In addition to the eight pre-registered subregions of SIIPS that are described in the main text, we also tested for effects in seven subregions that were not pre-registered but are of interest based on recent work. Six of these subregions–right nucleus accumbens (NAc), left and right dlPFC, right secondary somatosensory cortex (S2), right sensorimotor cortex (SMC) and left precuneus–are "suppressors", meaning that they are more active for higher stimulus intensities, but activity is associated with lower pain reports (i.e., in the original data based on which the SIIPS signature was developed [1] they had significant positive estimates for the effect of stimulus intensity, but

their weights in the SIIPS signature are negative). The last region–the vmPFC– is a mediator of pain, but with a negative sign: It de-activates with increasing stimulus intensity, and greater deactivation is associated with greater pain controlling for stimulus intensity.

For full statistics in these seven subregions for both modalities, see Supplementary Table 6 and Supplementary Table 7. Note that activity in SIIPS subregions represents the local pattern response, and thus (as explained in the main text) more positive responses indicate more pain-related activity in all regions. Following the findings from the previous study [1], we expected a significant positive effect of the stimulus intensity in the six suppressor regions, and a significant negative effect of stimulus intensity in the vmPFC. We further expected a significant placebo effect in all these seven regions, such that the pattern response score would be lower in the Placebo compared to the Control condition.

In the vmPFC, we found the expected significant negative effects of the stimulus intensity (not surviving Bonferroni correction) and the placebo treatment only in the mechanical modality. These effects were not significant in the thermal modality. In the suppressor regions, the placebo effect was significant only in the right NAc and left precuneus, and only in the mechanical modality. As for the stimulus level effect, it was not significantly positive in any of the suppressor regions. It was, however, unexpectedly significantly negative in the right S2 for both modalities, and in the right SMC and right dlPFC (the latter not surviving Bonferroni correction) only for the thermal modality.

<u>Supplementary Figures</u>

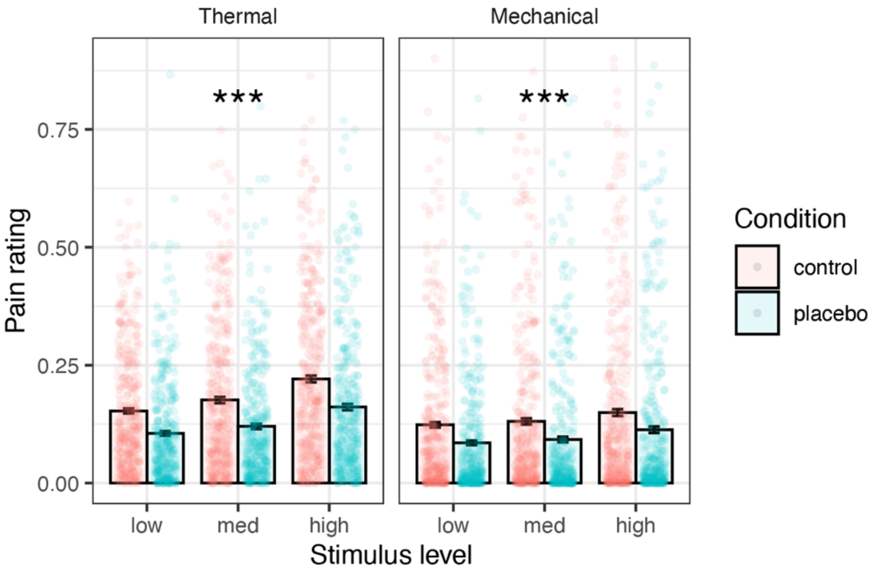

Supplementary Figure 1. Behavioral results. The mean pain ratings across participants are presented, in the control (red) and placebo (blue) condition, for each combination of modality and stimulus level. Error bars represent within-participant standard error of the mean, based on Morey, 2008 [2]. Points represent single participants (*N* = 374). Asterisks represent significance of the placebo effect (mixed-effects model, uncorrected): * $p < .05$, ** $p < .01$, *** $p < .001$.

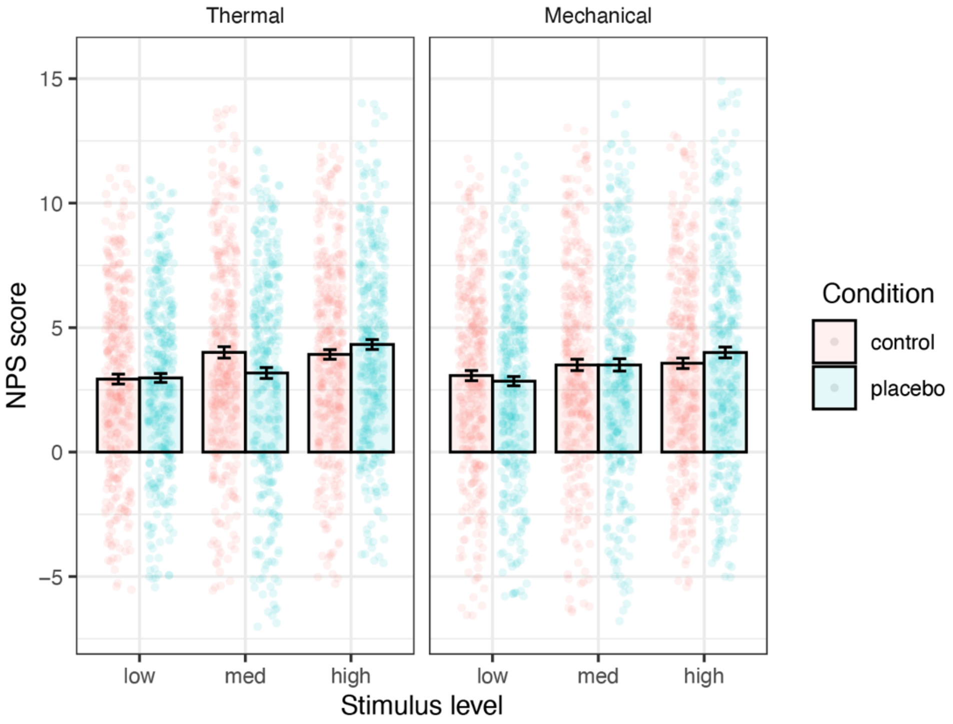

Supplementary Figure 2. NPS results. The mean NPS scores (based on dot product) across participants are presented, in the Control (red) and Placebo (blue) condition, for each combination of modality and stimulus level. Error bars represent within-participant standard error of the mean, based on Morey, 2008 [2]. Points represent single participants (N = 392). Note that for better visualization, the 2.5% lowest and 2.5% highest observations from each category were excluded (the bars and error bars are based on the entire distribution). Asterisks represent significance of the placebo effect (mixed-effects model, uncorrected): * p < .05, ** p < .01, *** p < .001.

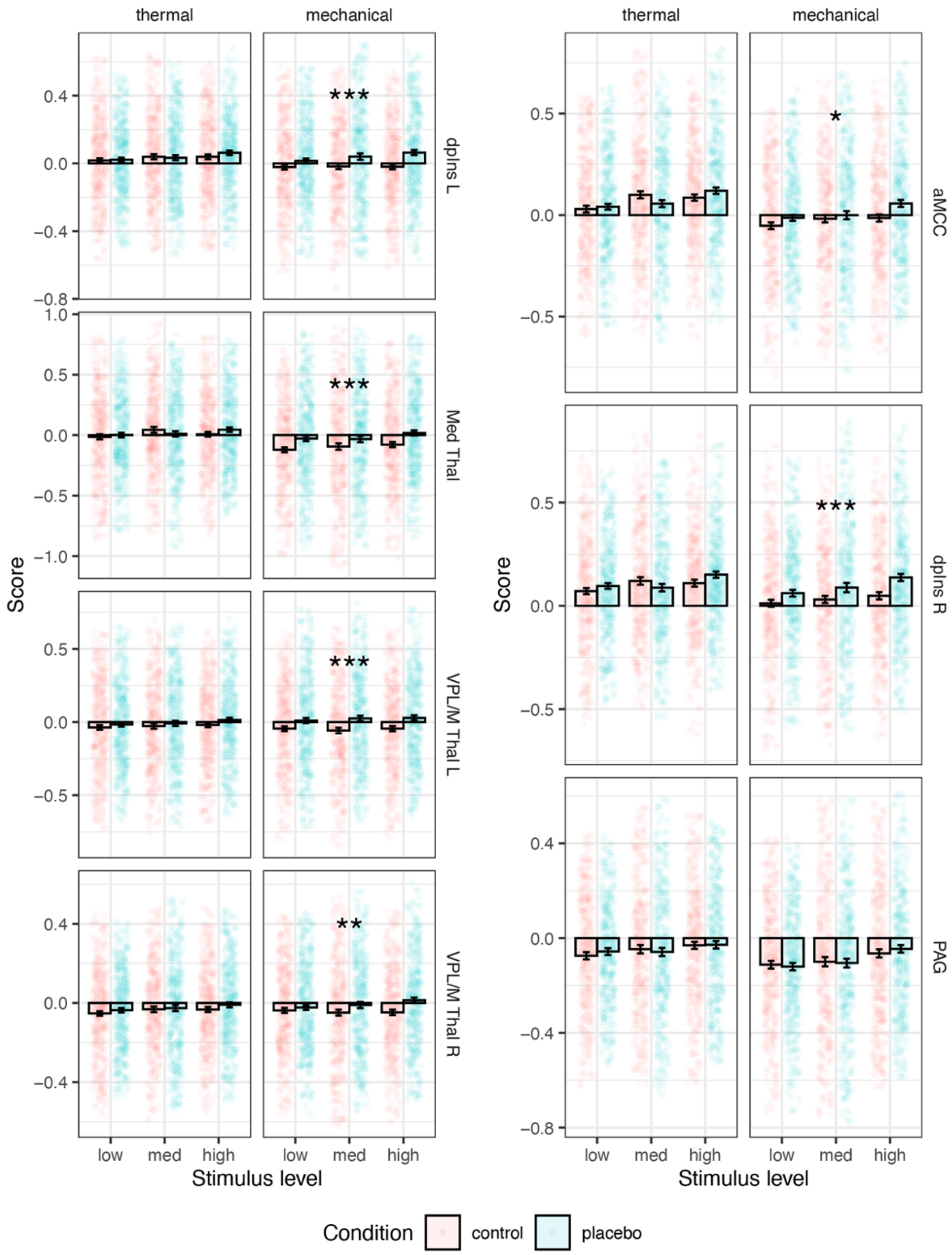

Supplementary Figure 3. Activity in a priori nociceptive regions of interest. The mean activity across voxels in each a priori nociceptive region across participants is presented, in the Control (red) and Placebo (blue) condition, for each combination of modality and stimulus level. Error bars represent within-participant standard error of the mean, based on Morey, 2008 [2]. Points represent single participants (N = 392). Note that for better visualization, the 5% lowest and 5% highest observations from each category were excluded (the bars and error bars are based on the entire distribution). Abbreviations: L (left), R (right), aMCC (anterior midcingulate cortex), dpIns (dorsal posterior insula); Med Thal (medial thalamus); VPL/M Thal (ventral posterior thalamus). Asterisks represent significance of the placebo effect (mixed-effects model, uncorrected): * $p < .05$, ** $p < .01$, *** $p < .001$.

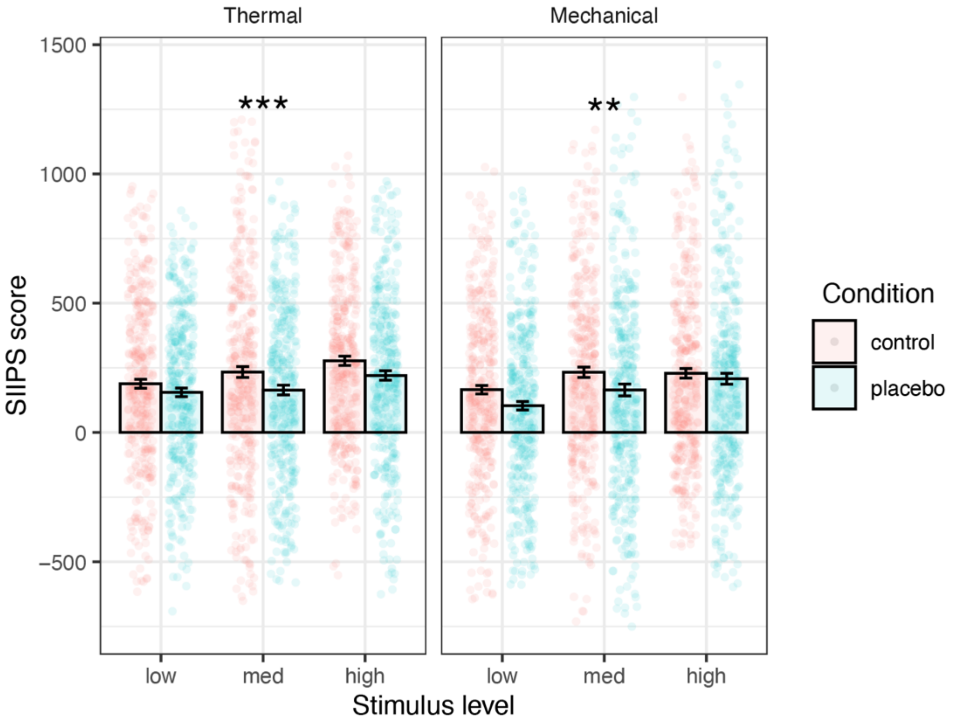

Supplementary Figure 4. SIIPS results. The mean SIIPS scores (based on dot product) across participants are presented, in the Control (red) and Placebo (blue) condition, for each combination of modality and stimulus level. Error bars represent within-participant standard error of the mean, based on Morey, 2008 [2]. Points represent single participants (*N* = 392). Note that for better visualization, the 2.5% lowest and 2.5% highest observations from each category were excluded (the bars and error bars are based on the entire distribution). Asterisks represent significance of the placebo effect (mixed-effects model, uncorrected): * $p < .05$, ** $p < .01$, *** $p < .001$.

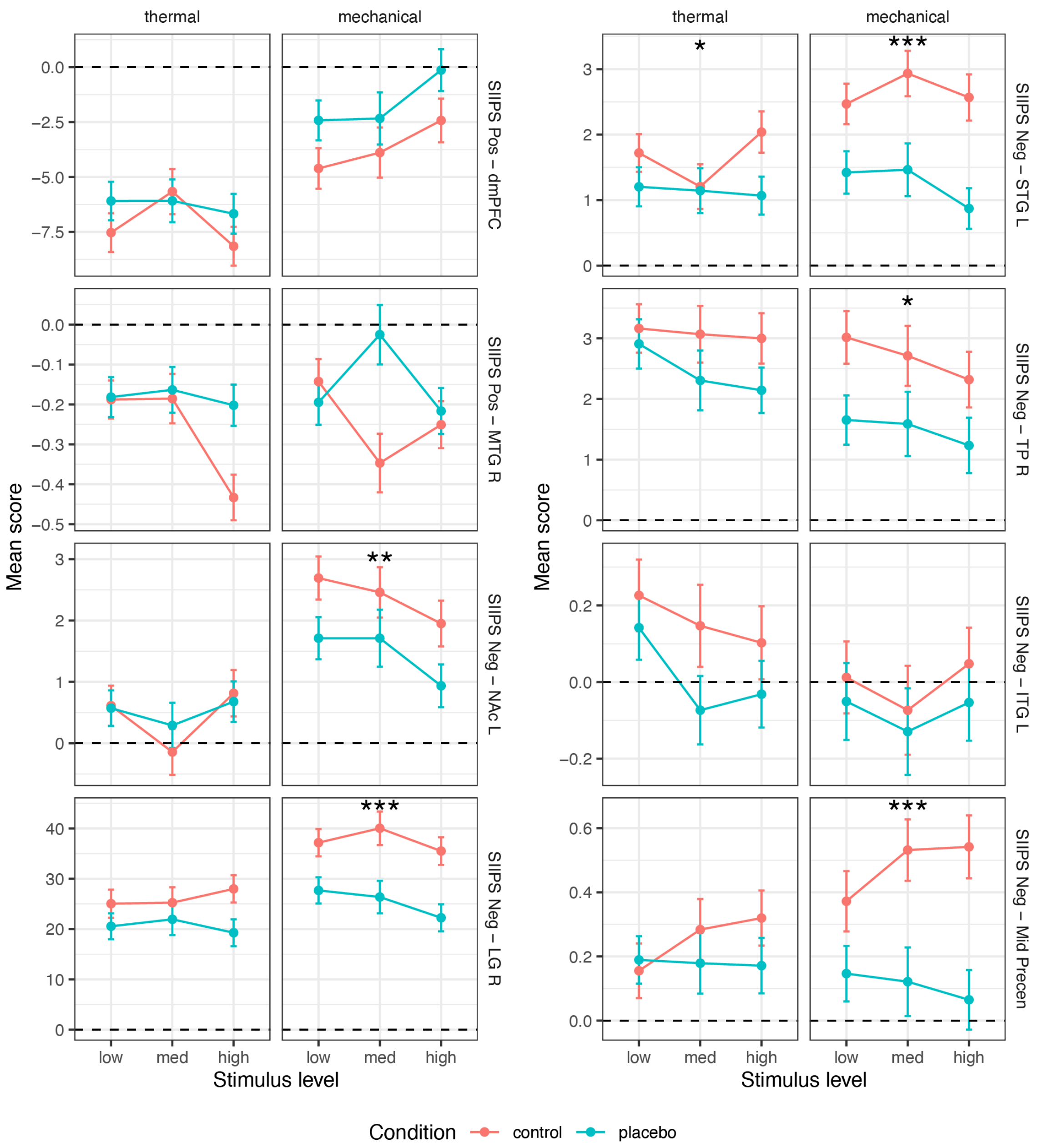

Supplementary Figure 5. Activity in a priori SIIPS subregions.

The local pattern responses of each a priori subregion of SIIPS across participants are presented, in the Control (red) and Placebo (blue) condition, for each combination of modality and stimulus level. Error bars represent within-participant standard error of the mean, based on Morey, 2008 [2]. *N* = 392 participants. Asterisks represent significance of the placebo effect (mixed-effects model, uncorrected): * $p < .05$, ** $p < .01$, *** $p < .001$. See also Supplementary Figure 6. Abbreviation: SIIPS (Stimulus Intensity Independent Pain Signature); Pos (positive); Neg (negative); L (left); R (right); dmPFC (dorsomedial prefrontal cortex); MTG (middle temporal gyrus); NAc (nucleus accumbens); LG (lingual gyrus); STG (superior temporal gyrus); TP (temporal pole); ITG (inferior temporal gyrus); mid precen (middle precentral gyrus).

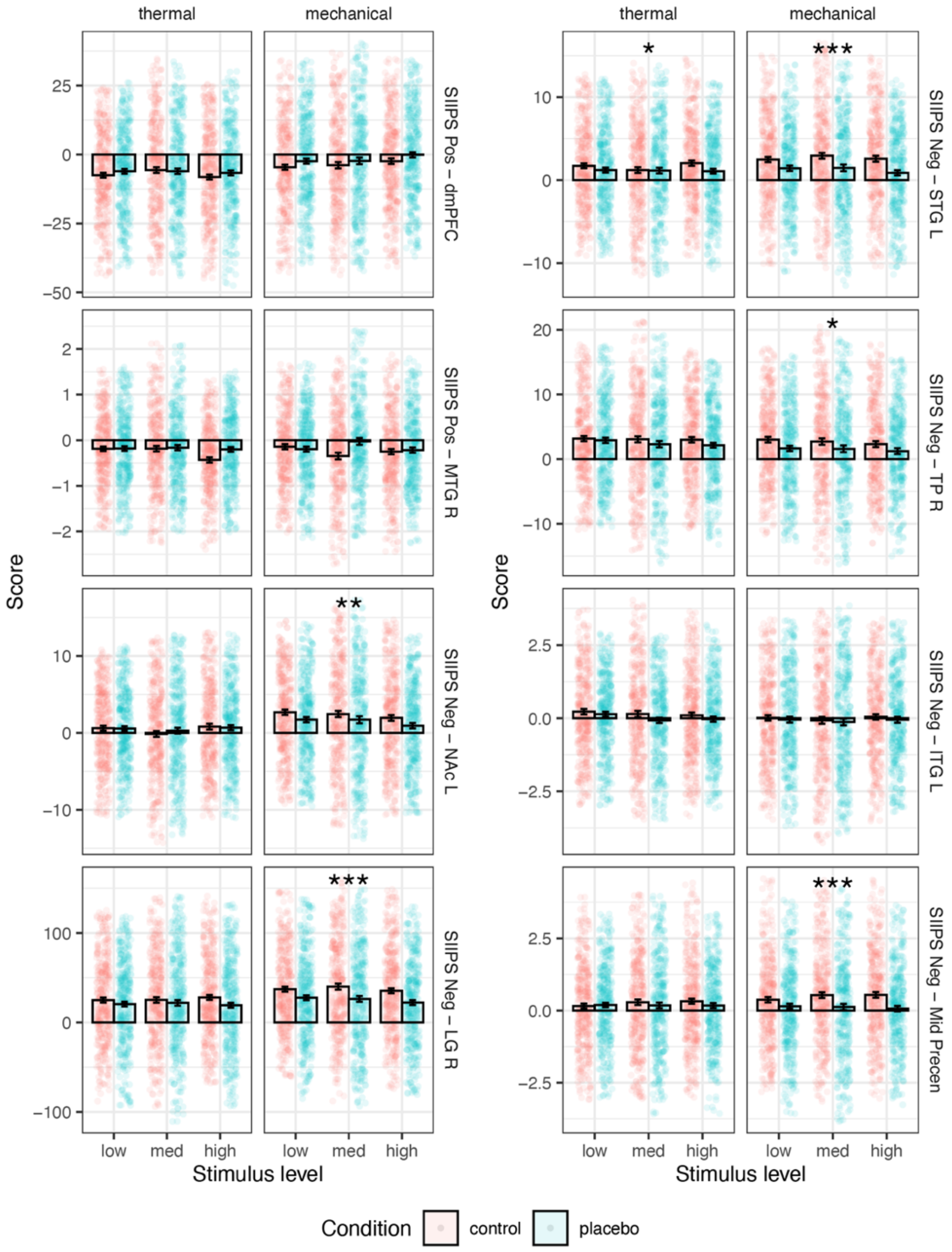

Supplementary Figure 6. Activity in a priori SIIPS subregions. The local pattern responses of each a priori subregion across participants is presented, in the Control (red) and Placebo (blue) condition, for each combination of modality and stimulus level. Error bars represent within-participant standard error of the mean, based on Morey, 2008 [2]. Points represent single participants (*N* = 392). Note that for better visualization, the 5% lowest and 5% highest observations from each category were excluded (the bars and error bars are based on the entire distribution). Asterisks represent significance of the placebo effect (mixed-effects model, uncorrected): * *p* < .05, ** *p* < .01, *** *p* < .001. Abbreviations: SIIPS (Stimulus Intensity Independent Pain Signature); Pos (positive); Neg (negative); L (left); R (right); dmPFC (dorsomedial prefrontal cortex); MTG (middle temporal gyrus); NAc (nucleus accumbens); LG (lingual gyrus); STG (superior temporal gyrus); TP (temporal pole); ITG (inferior temporal gyrus); mid precen (middle precentral gyrus).

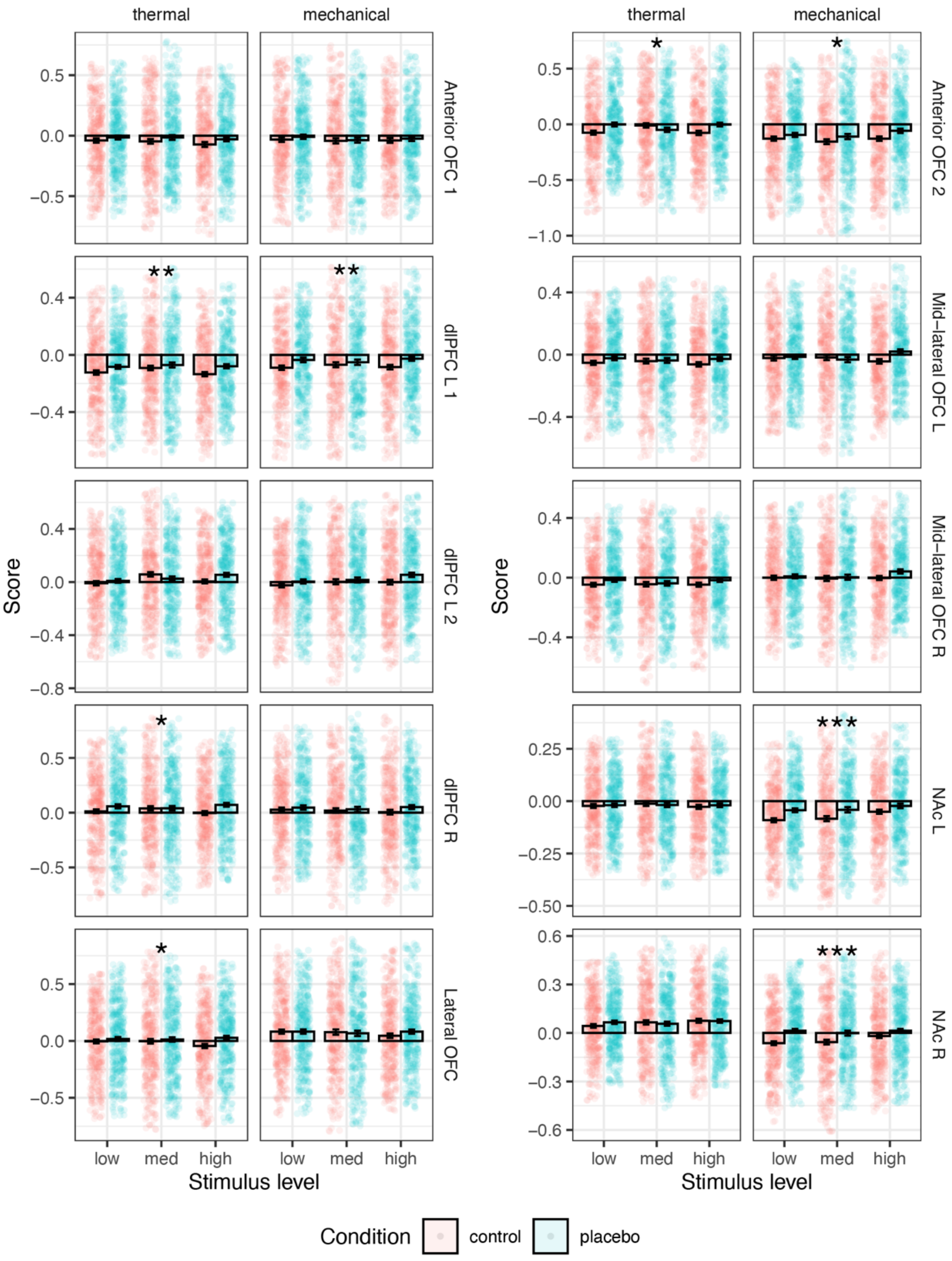

Supplementary Figure 7. Activity in a priori higher-level pain processing regions. The mean activity across voxels in each a priori ROI across participants is presented, in the Control (red) and Placebo (blue) condition, for each combination of modality and stimulus level. Error bars represent within-participant standard error of the mean, based on Morey, 2008 [2]. Points represent single participants (N = 392). Note that for better visualization, the 5% lowest and 5% highest observations from each category were excluded (the bars and error bars are based on the entire distribution). Asterisks represent significance of the placebo effect (mixed-effects model, uncorrected): * p < .05, ** p < .01, *** p < .001. Abbreviations: L (left); R (right); NAc (nucleus accumbens); dlPFC (dorsolateral prefrontal cortex); OFC (orbitofrontal cortex).

Supplementary Tables

Supplementary Table 1.

A priori pre-registered regions of interest.

Type and atlas on CANlab tools	Region full name	Region short name	Label (peak MNI coordinates)
Nociceptive (Pain pathways atlas; pain_pathways_atlas_obj.mat)	Anterior midcingulate cortex	aMCC	aMCC_MPFC
	Left dorsal posterior insula	Left dpIns	dpIns_L
	Right dorsal posterior insula	Right dpIns	dpIns_R
	Medial thalamus	Med Thal	Thal_MD
	Left ventral posterior thalamus	Left VPL/M Thal	Thal_VPLM_L
	Right ventral posterior thalamus	Right VPL/M Thal	Thal_VPLM_R
	Periaqueductal gray	PAG	Bstem_PAG
SIIPS subregions positive weights (nonnoc_v11_4_subcluster_maps_fdr05_pattern_wttest.nii)	Right dorsomedial prefrontal cortex	SIIPS Pos - dmPFC	dmPFC (12, 50, 40)
	Right middle temporal gyrus	SIIPS Pos - MTG R	R MTG (70, -20, -14)
SIIPS subregions negative weights (nonnoc_v11_4_subcluster_maps_fdr05_pattern_wttest.nii)	Left nucleus Accumbens	SIIPS Neg - NAc L	L NAc (-14,14,-14)
	Right lingual gyrus	SIIPS Neg - LG R	R LG (12, -78, -8)
	Left superior temporal gyrus	SIIPS Neg - STG L	L STG (-60, -30, 6)
	Right temporal pole	SIIPS Neg - TP R	R TP (32, 8, -40)
	Left inferior temporal gyrus	SIIPS Neg - ITG L	L ITG (-38, -4, -32)
	Middle precentral gyrus	SIIPS Neg - Mid Precen	Mid Precen (-6, -24, 52)
Higher-level (canlab2018_2mm)	Left dorsolateral prefrontal cortex area 1	Left dlPFC 1	Ctx_8C_L
	Left dorsolateral prefrontal cortex area 2	Left dlPFC 2	Ctx_46_L
	Right dorsolateral prefrontal cortex	Right dlPFC	Ctx_p9_46v_R
	Lateral orbitofrontal cortex	Lateral OFC	Ctx_a47r_R
	Anterior orbitofrontal cortex area 1	Anterior OFC 1	Ctx_a10p_R
	Anterior orbitofrontal cortex area 2	Anterior OFC 2	Ctx_p10p_R
	Right mid-lateral orbitofrontal cortex	Right mid-lateral OFC	Ctx_11l_R
	Left mid-lateral orbitofrontal cortex	Left mid-lateral OFC	Ctx_11l_L
	Right nucleus accumbens	Right NAc	V_Striatum_R
	Left nucleus accumbens	Left NAc	V_Striatum_L

Supplementary Table 2.

Bayes Factors for a priori nociceptive neuromarker and ROIs.

Modality	Model	NPS	Left dpIns	Right dpIns	aMCC	Left VPL/M Thal	Right VPL/M Thal	Medial Thal	PAG
Thermal	wide	0.044 (0.098)	0.037 (0.072)	0.048 (0.060)	0.034 (0.132)	0.143 (0.190)	0.086 (0.058)	0.033 (0.134)	0.033 (0.033)
	wide with interaction	0.056 (0.097)	0 (0.363)	0.004 (0.054)	0.004 (0.117)	0.001 (0.334)	0.001 (0.123)	0.001 (0.136)	0 (0.040)
	regular width	0.069 (0.234)	0.160 (0.641)	0.082 (0.126)	0.053 (0.215)	0.126 (0.411)	0.121 (0.076)	0.048 (0.113)	0.048 (0.032)
	ultrawide	0.036 (0.172)	0.027 (0.082)	0.034 (0.113)	0.020 (0.099)	0.102 (0.133)	0.057 (0.076)	0.025 (0.066)	0.025 (0.069)
Mechanical	wide	0.036 (0.117)	820.7 (0.226)	374.3 (0.234)	2 (0.081)	1125.3 (0.181)	5.9 (0.091)	394.4 (0.132)	0.032 (0.069)
	wide with interaction	0.001 (0.096)	8.5 (0.112)	3.1 (0.203)	0.036 (0.077)	7.5 (0.196)	0.110 (0.072)	2.547 (0.091)	0 (0.069)
	regular width	0.049 (0.071)	785.2 (0.071)	614.5 (0.378)	2.9 (0.091)	1674.3 (0.066)	8.3 (0.089)	412.2 (0.100)	0.048 (0.058)
	ultrawide	0.024 (0.050)	447.3 (0.134)	213 (0.108)	1.3 (0.087)	631 (0.464)	3.8 (0.083)	289.8 (0.196)	0.023 (0.057)

Bayes Factor (BF) values for tests of placebo effect in a priori nociceptive neuromarker and ROIs. BF values (evidence in favor of the alternative hypothesis divided by the evidence in favor of the null hypothesis) are presented, along with the corresponding error of the BF value in parenthesis, for each neuromarker / ROI, modality, width of the prior distribution ($\sqrt{2}/2$, 1, and $\sqrt{2}$) and inclusion of the interaction term. Abbreviations: aMCC (anterior midcingulate cortex), dpIns (dorsal posterior insula); thal (thalamus); VPL/M (ventral posterior thalamus); PAG (periaqueductal gray).

Supplementary Table 3.

Bayes Factors for a priori higher-level pain processing neuromarker and ROIs.

Modality	Model	SIIPS	Left dlPFC 1	Left dlPFC 2	Right dlPFC	Lateral OFC	Anterior OFC 1	Anterior OFC 2	Right mid-lateral OFC	Left mid-lateral OFC	Left NAc	Right NAc
Thermal	wide	93.9 (0.139)	5.7 (0.090)	0.055 (0.161)	0.853 (0.202)	0.633 (0.138)	0.354 (0.104)	0.543 (0.066)	0.225 (0.137)	0.263 (0.063)	0.037 (0.165)	0.037 (0.143)
	wide with interaction	1.1 (0.163)	0.047 (0.068)	0.010 (0.117)	0.034 (0.205)	0.014 (0.143)	0.003 (0.199)	1.8 (0.072)	0.002 (0.133)	0.002 (0.072)	0 (0.064)	0 (0.102)
	Regular width	168.8 (0.115)	7.5 (0.067)	0.067 (0.062)	1.6 (0.992)	0.969 (0.072)	0.547 (0.069)	0.837 (0.126)	0.319 (0.135)	0.361 (0.064)	0.052 (0.956)	0.051 (0.138)
	ultrawide	93.8 (0.134)	3.8 (0.087)	0.037 (0.066)	0.816 (0.097)	0.404 (0.090)	0.284 (0.072)	0.516 (0.104)	0.148 (0.081)	0.206 (0.109)	0.023 (0.055)	0.025 (0.063)
Mechanical	wide	30.4 (0.312)	9.2 (0.281)	0.480 (0.071)	0.055 (0.399)	0.035 (0.092)	0.046 (0.041)	2.6 (0.064)	0.088 (0.042)	0.113 (0.049)	396.3 (0.156)	16314.1 (0.922)
	wide with interaction	0.352 (0.090)	0.094 (0.198)	0.011 (0.505)	0 (0.404)	0 (0.102)	0 (0.044)	0.017 (0.044)	0.019 (0.236)	0.002 (0.069)	3.1 (0.158)	316.8 (0.921)
	Regular width	28.7 (0.098)	8.4 (0.063)	0.620 (0.066)	0.149 (0.067)	0.053 (0.096)	0.071 (0.055)	3.7 (0.174)	0.141 (0.056)	0.158 (0.053)	559.3 (0.049)	22611.1 (0.073)
	ultrawide	13.2 (0.131)	3.9 (0.121)	0.294 (0.092)	0.066 (0.043)	0.023 (0.072)	0.036 (0.091)	1.9 (0.055)	0.073 (0.072)	0.080 (0.062)	478.9 (0.423)	12963.3 (0.122)

Bayes Factor values for tests of placebo effect in a priori higher-level pain processing neuromarker and ROIs. BF values (evidence in favor of the alternative hypothesis divided by the evidence in favor of the null hypothesis) are presented, along with the corresponding error of the BF value in parenthesis, for each neuromarker / ROI, modality, width of the prior distribution ($\sqrt{2}/2$, 1, and $\sqrt{2}$) and inclusion of the interaction term. Abbreviations: dlPFC (dorso-lateral prefrontal cortex); OFC (orbitofrontal cortex); NAc (nucleus accumbens).

Supplementary Table 4. Statistics for a priori subregions of SIIPS: stimulus level effect.

Region	Modality	Estimate	95% CI	SE	t	DF	p
SIIPS Pos - dmPFC	Thermal	-0.028	[-0.106, 0.050]	0.040	-0.70	998.6	0.484
	Mechanical	0.089	[0.009, 0.168]	0.041	2.19	1194.3	**0.029**
SIIPS Pos - MTG R	Thermal	-0.108	[-0.197, -0.019]	0.045	-2.39	773.9	**0.017**
	Mechanical	-0.046	[-0.132, 0.040]	0.044	-1.06	1389.9	0.291
SIIPS Neg - NAc L	Thermal	0.023	[-0.065, 0.112]	0.045	0.52	810.0	0.604
	Mechanical	-0.089	[-0.179, 0.001]	0.046	-1.95	806.9	0.052
SIIPS Neg - LG R	Thermal	0.015	[-0.065, 0.094]	0.041	0.36	528.3	0.720
	Mechanical	-0.051	[-0.131, 0.029]	0.041	-1.25	664.7	0.214
SIIPS Neg - STG L	Thermal	0.012	[-0.071, 0.094]	0.042	0.28	690.3	0.782
	Mechanical	-0.031	[-0.113, 0.050]	0.042	-0.75	1213.8	0.453
SIIPS Neg - TP R	Thermal	-0.047	[-0.133, 0.039]	0.044	-1.07	1558.6	0.286
	Mechanical	-0.061	[-0.154, 0.033]	0.048	-1.27	341.9	0.203
SIIPS Neg - ITG L	Thermal	-0.069	[-0.152, 0.015]	0.042	-1.62	680.2	0.106
	Mechanical	0.009	[-0.079, 0.096]	0.045	0.20	1572.1	0.846
SIIPS Neg - Mid Precen	Thermal	0.034	[-0.045, 0.113]	0.040	0.84	848.9	0.401
	Mechanical	0.016	[-0.063, 0.096]	0.040	0.40	516.6	0.688

Full statistics for the mixed effects models of the activity in each a priori subregion of SIIPS, for the stimulus level (Stim level) effect. Abbreviations: CI (confidence interval); DF (degrees of freedom); Pos (positive); Neg (negative); L (left); R (right); dmPFC (dorsomedial prefrontal cortex); MTG (middle temporal gyrus); NAc (nucleus accumbens); LG (lingual gyrus); STG (superior temporal gyrus); TP (temporal pole); ITG (inferior temporal gyrus); mid precen (middle precentral gyrus). Significant p values (uncorrected p < .05) are marked in bold.

Supplementary Table 5. Statistics for a priori subregions of SIIPS: placebo effect.

Region	Modality	Estimate	95% CI	SE	t	DF	p
SIIPS Pos - dmPFC	Thermal	0.035	[-0.035, 0.105]	0.036	0.99	228.5	0.324
	Mechanical	0.078	[-0.003, 0.158]	0.041	1.91	230.9	0.058
SIIPS Pos - MTG R	Thermal	0.074	[-0.003, 0.152]	0.039	1.89	434.6	0.060
	Mechanical	0.065	[-0.012, 0.142]	0.039	1.66	325.2	0.098
SIIPS Neg - NAc L	Thermal	0.012	[-0.059, 0.083]	0.036	0.34	851.7	0.735
	Mechanical	-0.111	[-0.187, -0.034]	0.039	-2.85	466.6	**0.005**
SIIPS Neg - LG R	Thermal	-0.073	[-0.148, 0.002]	0.038	-1.93	211.4	0.055
	Mechanical	-0.165	[-0.238, -0.093]	0.037	-4.47	422.3	**< .001**
SIIPS Neg - STG L	Thermal	-0.069	[-0.135, -0.002]	0.034	-2.03	1145.0	**0.043**
	Mechanical	-0.161	[-0.231, -0.092]	0.035	-4.56	749.2	**< .001**
SIIPS Neg - TP R	Thermal	-0.064	[-0.138, 0.009]	0.037	-1.72	239.6	0.087
	Mechanical	-0.104	[-0.186, -0.021]	0.042	-2.48	226.5	**0.014**
SIIPS Neg - ITG L	Thermal	-0.066	[-0.136, 0.005]	0.036	-1.83	505.8	0.069
	Mechanical	-0.026	[-0.105, 0.052]	0.040	-0.66	214.9	0.511
SIIPS Neg - Mid Precen	Thermal	-0.029	[-0.095, 0.036]	0.033	-0.88	323.6	0.381
	Mechanical	-0.156	[-0.228, -0.085]	0.036	-4.30	386.6	**< .001**

Full statistics for the mixed effects models of the activity in each a priori subregion of SIIPS, for the placebo effect. Abbreviations: CI (confidence interval); DF (degrees of freedom); Pos (positive); Neg (negative); L (left); R (right); dmPFC (dorsomedial prefrontal cortex); MTG (middle temporal gyrus); NAc (nucleus accumbens); LG (lingual gyrus); STG (superior temporal gyrus); TP (temporal pole); ITG (inferior temporal gyrus); mid precen (middle precentral gyrus). Significant p values (uncorrected $p < .05$) are marked in bold. Negative coefficients indicate reductions with Placebo vs. Control, which was the expected direction of effects.

Supplementary Table 6.

Statistics for additional, non-pre-registered subregions of SIIPS: stimulus level effect.

Region	Modality	Estimate	95% CI	SE	t	DF	p
SIIPS Neg - vmPFC	Thermal	0.061	[-0.019, 0.141]	0.041	1.49	643	0.136
	Mechanical	-0.100	[-0.186, -0.013]	0.044	-2.25	1246.4	**0.024**
SIIPS Neg - NAc R	Thermal	-0.077	[-0.161, 0.007]	0.043	-1.79	1333.3	0.074
	Mechanical	-0.081	[-0.169, 0.006]	0.044	-1.83	1411.6	0.068
SIIPS Neg - dlPFC L	Thermal	-0.075	[-0.154, 0.005]	0.040	-1.85	707.3	0.065
	Mechanical	-0.037	[-0.117, 0.043]	0.041	-0.91	1380.2	0.361
SIIPS Neg - dlPFC R	Thermal	-0.086	[-0.160, -0.013]	0.038	-2.30	944.8	**0.022**
	Mechanical	-0.029	[-0.108, 0.049]	0.040	-0.73	573.6	0.465
SIIPS Neg - S2 R	Thermal	-0.115	[-0.190, -0.039]	0.038	-2.99	676.6	**0.003**
	Mechanical	-0.128	[-0.206, -0.050]	0.040	-3.21	351.8	**0.001**
SIIPS Neg - SMC R	Thermal	-0.151	[-0.229, -0.072]	0.040	-3.78	635.2	**<.001**
	Mechanical	-0.025	[-0.105, 0.055]	0.041	-0.60	569.3	0.547
SIIPS Neg - Precun L	Thermal	0.019	[-0.067, 0.105]	0.044	0.44	297.7	0.664
	Mechanical	-0.007	[-0.090, 0.076]	0.042	-0.17	432.5	0.867

Full statistics for the mixed effects models of the activity in each a priori subregion of SIIPS, for the stimulus level effect. Abbreviations: CI (confidence interval); DF (degrees of freedom); Neg (negative); L (left); R (right); vmPFC (ventromedial prefrontal cortex); dmPFC (dorsomedial prefrontal cortex); NAc (nucleus accumbens); S2 (secondary somatosensory cortex); SMC (sensorimotor cortex); precun (precuneus). Significant p values (uncorrected $p < .05$) are marked in bold.

Supplementary Table 7.

Statistics for additional, non-pre-registered subregions of SIIPS: placebo effect.

Region	Modality	Estimate	95% CI	SE	t	DF	p
SIIPS Neg - vmPFC	Thermal	-0.020	[-0.095, 0.055]	0.038	-0.53	297.0	0.597
	Mechanical	-0.164	[-0.244, -0.085]	0.041	-4.06	391.4	**<.001**
SIIPS Neg - NAc R	Thermal	0.062	[-0.016, 0.140]	0.040	1.56	314.3	0.120
	Mechanical	-0.114	[-0.189, -0.040]	0.038	-3.01	391.5	**0.003**
SIIPS Neg - dlPFC L	Thermal	-0.049	[-0.119, 0.021]	0.035	-1.39	453.6	0.167
	Mechanical	-0.039	[-0.112, 0.034]	0.037	-1.06	374.5	0.292
SIIPS Neg - dlPFC R	Thermal	-0.031	[-0.095, 0.033]	0.032	-0.96	354.2	0.339
	Mechanical	0.051	[-0.020, 0.122]	0.036	1.40	385.6	0.162
SIIPS Neg - S2 R	Thermal	0.032	[-0.040, 0.104]	0.037	0.87	226.8	0.385
	Mechanical	-0.029	[-0.104, 0.045]	0.038	-0.77	273.2	0.442
SIIPS Neg - SMC R	Thermal	0.034	[-0.035, 0.104]	0.035	0.97	563.5	0.331
	Mechanical	0.051	[-0.025, 0.128]	0.039	1.32	240.1	0.188
SIIPS Neg - Precun L	Thermal	-0.059	[-0.127, 0.010]	0.035	-1.69	316.9	0.092
	Mechanical	-0.151	[-0.226, -0.077]	0.038	-4.00	224.6	**<.001**

Full statistics for the mixed effects models of the activity in each a priori subregion of SIIPS, for the placebo effect. Abbreviations: CI (confidence interval); DF (degrees of freedom); Neg (negative); L (left); R (right); vmPFC (ventromedial prefrontal cortex); dmPFC (dorsomedial prefrontal cortex); NAc (nucleus accumbens); S2 (secondary somatosensory cortex); SMC (sensorimotor cortex); precun (precuneus). Significant p values (uncorrected *p* < .05) are marked in bold.

Supplementary Table 8.

Statistics for a priori nociceptive ROIs: correlation between behavioral and neural placebo-induced reductions.

Region	Modality	Estimate	95% CI	SE	t	DF	p
aMCC	Thermal	0.152	[0.084, 0.220]	0.034	4.47	88.0	**< .001**
	Mechanical	0.171	[0.093, 0.248]	0.039	4.38	94.3	**< .001**
dpIns L	Thermal	0.056	[-0.012, 0.124]	0.034	1.63	129.9	0.106
	Mechanical	0.142	[0.068, 0.215]	0.037	3.84	87.3	**< .001**
dpIns R	Thermal	0.093	[0.029, 0.157]	0.033	2.86	230.5	**0.005**
	Mechanical	0.136	[0.064, 0.208]	0.036	3.76	79.6	**< .001**
PAG	Thermal	0.100	[0.033, 0.167]	0.034	2.95	134.5	**0.004**
	Mechanical	0.167	[0.096, 0.239]	0.036	4.70	49.6	**< .001**
Med Thal	Thermal	0.083	[0.015, 0.151]	0.035	2.41	180.2	**0.017**
	Mechanical	0.065	[-0.006, 0.136]	0.036	1.81	219.3	0.071
VPL/M Thal L	Thermal	0.035	[-0.029, 0.099]	0.033	1.07	232.6	0.286
	Mechanical	0.051	[-0.023, 0.124]	0.037	1.36	127.9	0.178
VPL/M Thal R	Thermal	0.035	[-0.033, 0.103]	0.034	1.02	117.2	0.311
	Mechanical	0.054	[-0.013, 0.121]	0.034	1.58	144.1	0.116

Full statistics for the mixed effects models of the correlation of the Control - Placebo neural activity in each a priori ROI and the behavioral analgesia (Control - Placebo pain ratings). Abbreviations: CI (confidence interval); DF (degrees of freedom); L (left); R (right); aMCC (anterior midcingulate cortex); dpIns (dorsal posterior insula); Med Thal (medial thalamus); VPL/M Thal (ventral posterior thalamus); PAG (periaqueductal gray). Significant p values (uncorrected $p < .05$) are marked in bold.

Supplementary Table 9.

Statistics for a priori subregions of SIIPS: correlation between behavioral and neural placebo-induced reductions.

Region	Modality	Estimate	95% CI	SE	t	DF	p
SIIPS Pos - dmPFC	Thermal	0.023	[-0.042, 0.087]	0.033	0.70	272.7	0.485
	Mechanical	0.072	[-0.004, 0.148]	0.038	1.88	89	0.064
SIIPS Pos - MTG R	Thermal	-0.020	[-0.087, 0.047]	0.034	-0.59	226.3	0.555
	Mechanical	-0.005	[-0.072, 0.061]	0.034	-0.16	171.3	0.873
SIIPS Neg - NAc L	Thermal	-0.041	[-0.105, 0.022]	0.032	-1.28	189.5	0.203
	Mechanical	-0.030	[-0.104, 0.045]	0.037	-0.79	71.2	0.430
SIIPS Neg - LG R	Thermal	0.093	[0.029, 0.157]	0.032	2.88	199.5	**0.004**
	Mechanical	-0.011	[-0.081, 0.058]	0.035	-0.32	106.5	0.747
SIIPS Neg - STG L	Thermal	0.011	[-0.054, 0.076]	0.033	0.33	128.8	0.743
	Mechanical	-0.091	[-0.158,-0.024]	0.034	-2.70	120	**0.008**
SIIPS Neg - TP R	Thermal	0.023	[-0.047, 0.093]	0.035	0.65	146	0.519
	Mechanical	-0.079	[-0.144,-0.013]	0.033	-2.35	218.3	**0.020**
SIIPS Neg - ITG L	Thermal	0.035	[-0.029, 0.098]	0.032	1.08	391.6	0.282
	Mechanical	-0.022	[-0.088, 0.044]	0.033	-0.66	199.9	0.508
SIIPS Neg - Mid Precen	Thermal	0.029	[-0.034, 0.091]	0.032	0.90	354.4	0.368
	Mechanical	-0.028	[-0.098, 0.043]	0.035	-0.78	90.2	0.438

Full statistics for the mixed effects models of the correlation of the Control - Placebo neural activity in each a priori subregion and the behavioral analgesia (Control - Placebo pain ratings). Abbreviations: CI (confidence interval); DF (degrees of freedom); Pos (positive); Neg (negative); L (left); R (right); dmPFC (dorsomedial prefrontal cortex); MTG (middle temporal gyrus); NAc (nucleus accumbens); LG (lingual gyrus); STG (superior temporal gyrus); TP (temporal pole); ITG (inferior temporal gyrus); mid precen (middle precentral gyrus). Significant p values (uncorrected $p < .05$) are marked in bold.

Supplementary Table 10.

Statistics for a priori ROIs for higher-level pain processing: correlation between behavioral and neural placebo-induced reductions.

Region	Modality	Estimate	95% CI	SE	t	DF	p
dlPFC L 1	Thermal	0.033	[-0.032, 0.097]	0.033	0.99	221	0.323
	Mechanical	0.038	[-0.030, 0.105]	0.034	1.11	238.3	0.269
dlPFC L 2	Thermal	0.081	[0.016, 0.145]	0.033	2.46	269.6	**0.014**
	Mechanical	0.087	[0.014, 0.161]	0.037	2.36	108.3	**0.020**
dlPFC R	Thermal	0.102	[0.040, 0.164]	0.032	3.23	243.5	**0.001**
	Mechanical	0.043	[-0.029, 0.115]	0.036	1.19	142.6	0.237
Lateral OFC	Thermal	0.031	[-0.032, 0.094]	0.032	0.96	328.4	0.341
	Mechanical	0.003	[-0.063, 0.070]	0.034	0.10	121.9	0.923
Anterior OFC 1	Thermal	0.022	[-0.042, 0.085]	0.032	0.67	513.6	0.504
	Mechanical	0.038	[-0.031, 0.107]	0.035	1.09	183.1	0.276
Anterior OFC 2	Thermal	0.027	[-0.035, 0.088]	0.031	0.85	836.3	0.394
	Mechanical	0.041	[-0.034, 0.115]	0.038	1.07	169.7	0.287
Mid-lateral OFC L	Thermal	-0.008	[-0.080, 0.064]	0.036	-0.22	178.4	0.827
	Mechanical	0.051	[-0.015, 0.116]	0.033	1.52	172.1	0.131
Mid-lateral OFC R	Thermal	0.015	[-0.057, 0.086]	0.036	0.40	164.6	0.688
	Mechanical	0.034	[-0.026, 0.094]	0.031	1.10	867.8	0.270
NAc L	Thermal	-0.034	[-0.096, 0.028]	0.031	-1.09	179.1	0.278
	Mechanical	0.065	[-0.002, 0.133]	0.034	1.91	150.8	0.058
NAc R	Thermal	0.068	[0.007, 0.129]	0.031	2.18	991.1	**0.030**
	Mechanical	0.072	[0.000, 0.145]	0.037	1.97	140.2	0.051

Full statistics for the mixed effects models of the correlation of the Control - Placebo neural activity in each a priori ROI and the behavioral analgesia (Control - Placebo pain ratings). Abbreviations: CI (confidence interval); DF (degrees of freedom); Pos (positive); Neg (negative); L (left); R (right); dlPFC (dorsolateral prefrontal cortex); OFC (orbitofrontal cortex); NAc (nucleus accumbens). Significant p values (uncorrected $p < .05$) are marked in bold.

<u>References</u>

1. Woo, C.-W., Chang, L. J., Lindquist, M. A. & Wager, T. D. Building better biomarkers: brain models in translational neuroimaging. *Nat. Neurosci.* **20**, 365–377 (2017).

2. Morey, R. D. Confidence Intervals from Normalized Data: a correction to Cousineau (2005). *Tutor. Quant. Methods Psychol.* **4**, 61–64 (2008).
