# Supplementary material for: Placebo treatment affects brain systems related to affective and cognitive processes, but not nociceptive pain
Source: Nat Commun. 2024 Jul 17;15:6017. doi: 10.1038/s41467-024-50103-8 (PMC11255344; doi:10.1038/s41467-024-50103-8)
Supplement: Supplementary file 3 — Reporting Summary [file 41467_2024_50103_MOESM3_ESM.pdf]

nature portfolio

Corresponding author(s): Tor Wager
Rotem Botvinik-Nezer

Last updated by author(s): Jun 14, 2024

Reporting Summary

Nature Portfolio wishes to improve the reproducibility of the work that we publish. This form provides structure for consistency and transparency in reporting. For further information on Nature Portfolio policies, see our Editorial Policies and the Editorial Policy Checklist.

Statistics

For all statistical analyses, confirm that the following items are present in the figure legend, table legend, main text, or Methods section.

n/a	Confirmed	
☐	☒	The exact sample size (*n*) for each experimental group/condition, given as a discrete number and unit of measurement
☐	☒	A statement on whether measurements were taken from distinct samples or whether the same sample was measured repeatedly
☐	☒	The statistical test(s) used AND whether they are one- or two-sided<br>*Only common tests should be described solely by name; describe more complex techniques in the Methods section.*
☐	☒	A description of all covariates tested
☐	☒	A description of any assumptions or corrections, such as tests of normality and adjustment for multiple comparisons
☐	☒	A full description of the statistical parameters including central tendency (e.g. means) or other basic estimates (e.g. regression coefficient) AND variation (e.g. standard deviation) or associated estimates of uncertainty (e.g. confidence intervals)
☐	☒	For null hypothesis testing, the test statistic (e.g. *F*, *t*, *r*) with confidence intervals, effect sizes, degrees of freedom and *P* value noted<br>*Give P values as exact values whenever suitable.*
☐	☒	For Bayesian analysis, information on the choice of priors and Markov chain Monte Carlo settings
☒	☐	For hierarchical and complex designs, identification of the appropriate level for tests and full reporting of outcomes
☐	☒	Estimates of effect sizes (e.g. Cohen's *d*, Pearson's *r*), indicating how they were calculated

Our web collection on statistics for biologists contains articles on many of the points above.

Software and code

Policy information about availability of computer code

Data collection	Data were collected with Matlab version 2018b.
Data analysis	MRI data were preprocessed with fMRIPrep version 20.2.3. fMRI GLM analysis was performed with Matlab version 2021b and SPM12 v7771. Further data analysis was performed with Matlab version 2020a and CANlab neuroimaging analysis tools (shared via Github at https://canlab.github.io/ neuroimaging analysis tools). For the Bayes Factor analysis, we used R version 4.2.2 with the BayesFactor package version 0.9.12-2. Analysis code are shared via Github: https://github.com/rotemb9/paingen-placebo-fmri-paper (release 2.0.0).

For manuscripts utilizing custom algorithms or software that are central to the research but not yet described in published literature, software must be made available to editors and reviewers. We strongly encourage code deposition in a community repository (e.g. GitHub). See the Nature Portfolio guidelines for submitting code & software for further information.

Data

Policy information about availability of data

All manuscripts must include a data availability statement. This statement should provide the following information, where applicable:

- Accession codes, unique identifiers, or web links for publicly available datasets
- A description of any restrictions on data availability
- For clinical datasets or third party data, please ensure that the statement adheres to our policy

Data are openly shared on OpenNeuro: ds004746; doi:10.18112/openneuro.ds004746.v1.0.0.

Research involving human participants, their data, or biological material

Policy information about studies with human participants or human data. See also policy information about sex, gender (identity/presentation), and sexual orientation and race, ethnicity and racism.

Reporting on sex and gender	We collected information about the sex of the participants (based on self reports). Sex-based analyses are not the main focus of the paper, but we report in the supplementary materials analyses accounting for sex (as a covariate) and a comparison between females and males. The sample included 231 women and 164 men.
Reporting on race, ethnicity, or other socially relevant groupings	We did not use race or ethnicity information in our analyses.
Population characteristics	Participants' age ranged from 30 to 43 years (M = 35.43, SD = 2.60).
Recruitment	Participants were recruited by telephone from the Colorado Community Twin Sample, which is derived from the Colorado Twin Registry, a population-based registry which has been run by the Institute of Behavioral Genetics (IBG) at the University of Colorado since 1984.
Ethics oversight	The experiment was approved by the institutional review board of the University of Colorado Boulder.

Note that full information on the approval of the study protocol must also be provided in the manuscript.

Field-specific reporting

Please select the one below that is the best fit for your research. If you are not sure, read the appropriate sections before making your selection.

☐ Life sciences ☒ Behavioural & social sciences ☐ Ecological, evolutionary & environmental sciences

For a reference copy of the document with all sections, see nature.com/documents/nr-reporting-summary-flat.pdf

Behavioural & social sciences study design

All studies must disclose on these points even when the disclosure is negative.

Study description	Experimental design with quantitative data.
Research sample	The study included a genetic component (which is beyond the scope of the current paper). Participants were recruited by telephone from the Colorado Community Twin Sample, which is derived from the Colorado Twin Registry, a population-based registry which has been run by the Institute of Behavioral Genetics (IBG) at the University of Colorado since 1984.
Sampling strategy	All participants included in the Colorado Community Twin Sample were contacted. The current paper is based on a pre-registered sample of 397 participants. This sample was pre-registered during data collection of a larger sample, and includes all participants who have been collected and at least minimally preprocessed (i.e., run through fmriprep) as of 09/01/2022 (a few days before the preregistration was submitted).
Data collection	Data were collected on a computer and with an MRI scanner. All conditions are within-participant. Experimenters were not blinded to the experimental conditions.
Timing	The first participant included in the study sample participated on November 18th, 2018, and the last one participated on August 2nd, 2022.
Data exclusions	Two participants who were included in the preregistered sample were excluded from analyses because of corrupted behavioral data. The dataset included 395 participants. As preregistered, individual stimulus trials were excluded if pain intensity rating response times were above 5.01 or below 0.02 seconds. These indicate either that the participant did not respond within the time allotted (five seconds) or responded too quickly to represent deliberate ratings. Entire participants were dropped if after dropping trials or scans due to above exclusion criteria, they lacked complete sets of conditions (placebo-thermal, placebo-mechanical, control-thermal, control-mechanical) for more than one stimulus level. This resulted in the exclusion of 3 additional participants (all males).
Non-participation	Until the preregistration, 32 participants started but did not complete the experiment: 18 due to technical issues, 8 due to physical constraints (related to the MRI scanner), and 6 participants chose to withdraw before completing the experiment.
Randomization	All conditions were within-participant (i.e., no group assignment).

Reporting for specific materials, systems and methods

We require information from authors about some types of materials, experimental systems and methods used in many studies. Here, indicate whether each material, system or method listed is relevant to your study. If you are not sure if a list item applies to your research, read the appropriate section before selecting a response.

Materials & experimental systems

n/a	Involved in the study
☒	☐ Antibodies
☒	☐ Eukaryotic cell lines
☒	☐ Palaeontology and archaeology
☒	☐ Animals and other organisms
☒	☐ Clinical data
☒	☐ Dual use research of concern
☒	☐ Plants

Methods

n/a	Involved in the study
☒	☐ ChIP-seq
☒	☐ Flow cytometry
☐	☒ MRI-based neuroimaging

Plants

Seed stocks: Not applicable

Novel plant genotypes: Not applicable

Authentication: Not applicable

Magnetic resonance imaging

Experimental design

Design type: Task fMRI, event-related

Design specifications: Each participant completed 4 runs of the placebo task. Each run consisted of 8 trials. Each trial consisted of 2 or 4 seconds of fixation, followed by a "get ready!" cue lasting 1 second, fixation for 1 or 3 seconds, stimulation (thermal/ mechanical pain) for 12 seconds, another fixation for 3 or 7 seconds, and two rating periods (intensity and unpleasantness) of 5 seconds each.

Behavioral performance measures: We recorded participants' ratings of intensity and unpleasantness of each stimulus, along with their response time (RT). RTs were used to exclude trials in which participants did not respond in time (RT > 5 seconds) or responded too quickly to represent deliberate ratings (RT < 0.02 seconds).

Acquisition

Imaging type(s): Functional and structural

Field strength: 3T

Sequence & imaging parameters: Imaging data were acquired using a 3T Siemens Prisma MRI scanner with a 32-channels head coil, at the University of Colorado at Boulder.
Structural: T1-weighted structural scan was acquired using a magnetization prepared rapid gradient echo (MPRAGE) pulse sequence with parallel imaging factor (iPAT) of 3, TR=2000ms, TE=2.11ms, flip angle=8 degrees, FOV=256mm, resolution=0.8×0.8×0.8mm.
Field maps: One with a posterior-anterior (PA) and one with an anterior-posterior (AP) direction, with the following imaging parameters: TR=7220ms, TE =73ms, flip angle=90 degrees, FOV=220mm, and in plane resolution of 2.7×2.7 X 2.7mm.
Functional: Four runs of the pain test task, acquired using T2*-weighted echo-planar imaging (EPI) sequence with multiband acceleration factor of 8, TR=460ms, TE=27.20ms, flip angle=44 degrees, FOV=220mm, resolution of 2.7× 2.7 X 2.7mm, 56 slices, and 550 acquired volumes per run.

Area of acquisition: Whole brain scan

Diffusion MRI: ☐ Used ☒ Not used

Preprocessing

Preprocessing software	Structural and functional data were preprocessed using fMRIPrep version 20.2.3 (RRID:SCR_016216).
Normalization	The single-band reference (SBRef) was used as a reference volume along with its skull-stripped version. A B0-nonuniformity map (or fieldmap) was estimated based on two EPI references with opposing phase-encoding directions, with 3dQwarp (AFNI 20160207). Based on the estimated susceptibility distortion, a corrected EPI reference was calculated for a more accurate co-registration with the anatomical reference. The BOLD reference was then co-registered to the T1w reference using bbregister (FreeSurfer) which implements boundary-based registration. Co-registration was configured with six degrees of freedom. The BOLD time-series were resampled into standard space, generating a preprocessed BOLD run in MNI152NLin2009cAsym space.
Normalization template	MNI152NLin2009cAsym
Noise and artifact removal	Nuisance regressors included 24 motion regressors (six motion parameters–translation and rotation in three directions–together with their derivatives, quadratics and derivatives of the quadratics) and a mean CSF signal regressor (estimated by fMRIPrep during preprocessing).
Volume censoring	We implemented spike censoring, with spikes identified by our in-house spike detection algorithm implemented by CanlabCore/diagnostics/scn_session_spike_id.m (available at github.com/canlab/CanlabCore).

Statistical modeling & inference

Model type and settings	We first performed whole-brain first level analysis at the subject level with a GLM model with regressors for each condition (combinations of pain modality [thermal/mechanical], condition [placebo/control], and intensity [low/medium/high]). This procedure resulted in 12 separate parameter estimate maps for each stimulus condition (e.g., high intensity thermal stimuli delivered to the placebo skin site), for each participant. Then, we computed the activity in each tested a priori region of interest or neuromarker for each condition of each participant, as a continuous score. These scores were then used in mixed-effects models, testing the effects of interest and accounting for the familial structure of our sample.
Effect(s) tested	We used mixed-effects models to test the following effects: Placebo (placebo vs. control condition), stimulus intensity, condition X intensity interaction, correlation between behavioral and neural placebo-induced reductions, and differences and correlations between modalities (thermal/mechanical pain).

Specify type of analysis: ☐ Whole brain ☒ ROI-based ☐ Both

Anatomical location(s)	We tested both a-priori neural markers and regions of interest, that were preregistered. Supplementary Table 1 lists all the regions along with the exact atlas and atlas label they were based on.

Statistic type for inference<br>(See Eklund et al. 2016)	Inferences were made on continuous scores per neuromarker / region, as described above.
Correction	Because false negatives and false positives here were equally important, we report results based on p < .05 without correcting for multiple comparisons across regions. This is because of the nature of the current study, focusing largely on testing pre-registered neural signatures and regions identified in previous literature regions with a substantially larger sample size. Nevertheless, to be slightly more conservative, we note in the paper when a result does not survive Bonferroni correction within its set of regions (e.g., within the set of seven nociceptive regions).

Models & analysis

n/a	Involved in the study
☒	☐ Functional and/or effective connectivity
☒	☐ Graph analysis
☐	☒ Multivariate modeling or predictive analysis

Multivariate modeling and predictive analysis	We used a-priori neuromarkers that were previously developed with multivariate modeling.
